# Inventory of a Neurological Intensive Care Unit: Who Is Treated and How Long?

**DOI:** 10.1155/2015/696038

**Published:** 2015-06-24

**Authors:** Roland Backhaus, Franz Aigner, Felix Schlachetzki, Dagmar Steffling, Wolfgang Jakob, Andreas Steinbrecher, Bernhard Kaiser, Peter Hau, Sandra Boy, Kornelius Fuchs, Ulrich Bogdahn, Markus Ritzka

**Affiliations:** ^1^Department of Neurology, University of Regensburg, 93053 Regensburg, Germany; ^2^Department of Anaesthesiology, HELIOS Klinikum, 99089 Erfurt, Germany; ^3^Department of Neurology, HELIOS Klinikum, 99089 Erfurt, Germany; ^4^Department of Surgery, University Clinic Regensburg, 93053 Regensburg, Germany

## Abstract

*Purpose*. To characterize indications, treatment, and length of stay in a stand-alone neurological intensive care unit with focus on comparison between ventilated and nonventilated patient. *Methods*. We performed a single-center retrospective cohort study of all treated patients in our neurological intensive care unit between October 2006 and December 2008. *Results*. Overall, 512 patients were treated in the surveyed period, of which 493 could be included in the analysis. Of these, 40.8% had invasive mechanical ventilation and 59.2% had not. Indications in both groups were predominantly cerebrovascular diseases. Length of stay was 16.5 days in mean for ventilated and 3.6 days for nonventilated patient. *Conclusion*. Most patients, ventilated or not, suffer from vascular diseases with further impairment of other organ systems or systemic complications. Data reflects close relationship and overlap of treatment on nICU with a standardized stroke unit treatment and suggests, regarding increasing therapeutic options, the high impact of acute high-level treatment to reduce consequential complications.

## 1. Introduction

The ageing society and a steadily increasing number of patients but limited financial resources challenges neurological intensive care medicine with its improving diagnostic and therapeutic possibilities. Hospital facilities need to adapt to these conditions. In 2007, roughly 500 beds for neurological intensive care were made available in Germany [1]. Although treatment of neurocritically ill patients in a specialized neurological intensive care unit (nICU) proved beneficial [2, 3], many of these beds are integrated in general ICUs, making specific neurological data on indication, treatment, and outcome difficult to obtain. To overcome these difficulties, the current study is based on data gathered in nICU in a “stand-alone” situation. The main university hospital hosts 7 additional ICUs of other departments including the neurosurgical ICU. The distances of about 2 km leads to a low rate of interhospital transfers and results in a well-defined neurological study population.

With regard to ventilated patients, neurological evaluation is considerably difficult and noninvasive diagnostics like ultrasound, neurophysiology (i.e., evoked potential, electroencephalography) are important instruments next to neurological know-how to monitor clinical developments. Neurological and cardiopulmonary surveillance, extensive diagnostics, and artificial ventilation including the weaning process are prime reasons for often prolonged nICU stay. Thus, the decision on tracheostomy and its best time point are daily and complex questions in clinical routine [4].

The aim of the study is to characterize diagnosis, treatment, and length of stay of patients within the context of a neurological intensive care unit.

## 2. Methods

### 2.1. Study Design

This descriptive retrospective study recorded indications, length of stay (LOS), diagnostic procedures, and complications for all patients treated in nICU of the Department of Neurology at University Hospital of Regensburg, Germany, between October 2006 and December 2008. Included were all patients primarily admitted to our nICU with a prima vista indication for neurological treatment or expected complication as a reason for treatment on nICU. We analyzed intensive care unit stay and process by file, characterized the treated patients, and compared between ventilated and nonventilated patients. The ethics commission of the University of Regensburg approved the study.

### 2.2. Basic Data and Classification by Diagnosis

Final diagnosis, ventilation, age at admission, length of intensive care unit stay, relevant complications, and number of procedures (cerebral computed tomography, computed tomographic angiography, thoracic computed tomography, magnetic resonance imaging (MRI), electroencephalography (EEG), electrophysiological examination, Doppler/Duplex-sonography, and echocardiography) were registered during the stay at the intensive care unit. Indications for nICU treatment are given for patients suffering from an acute or expectable cardiopulmonary instability as a consequence of a primary neurological disease.

**Figure 1:**
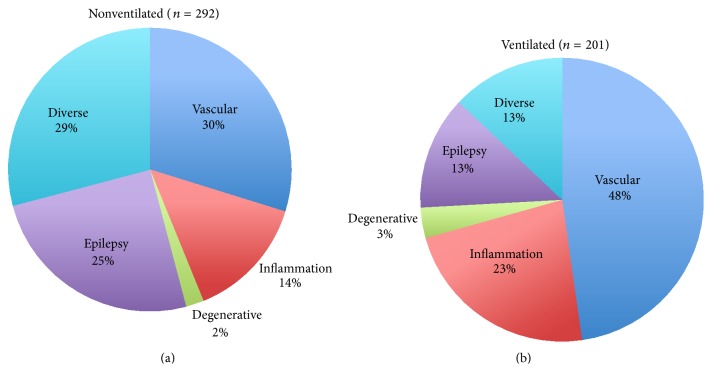
Indications for treatment on nICU.

The patients were classified into the following groups (Figures 1(a) and 1(b)):

1. vascular diseases (e.g., cerebral infarction/hemorrhage),
2. inflammatory diseases (e.g., meningitis, encephalitis, and Guillain-Barré Syndrome),
3. degenerative diseases (primarily Parkinson's disease),
4. neuromuscular disease (Lambert- Eaton Syndrome, Myasthenia gravis),
5. epilepsy,
6. other diseases (aggravation of neurological diseases due to nonneurological reasons such as dehydration or pneumonia, but also intoxications, septic encephalopathy, and others).

## 3. Results

Within the surveyed period, overall 512 patients were treated in the nICU. Complete datasets were available in 493 patients. Of these remaining 493 patients, 201 patients (40.8%) were ventilated mechanically. 47% of the patients were female; the mean age was 58 years. The average LOS on nICU for nonventilated patients was 3.6 days (standard deviation 0.5). For ventilated patients, the mean length of stay was 16.5 days. For both groups, ventilated and nonventilated patients, cerebrovascular diseases were diagnosed most frequently (ventilated: 47.8%, nonventilated: 23.6%). In the groups of patients with inflammatory and degenerative disease as an indication of nICU treatment ventilated and nonventilated patients are similarly distributed. Patients suffering from epilepsy did not have to be ventilated in most cases (5.5% versus 25%). A detailed cross tabulation of disease category and age for ventilated and nonventilated patients is given in Table 1.  Table 2 shows furthermore length of stay and procedures per patients and day.

### 3.1. Group Distribution

With regard to the group of nonventilated patients, Table 1 shows that 87 (29.8%) patients suffered from cerebrovascular diseases. Of this subsample, 21 patients (24.1%) were diagnosed with ischemic lesions of the anterior circulation, 10 (11.5%) with ischemic lesions of the posterior circulation, 20 (23.0%) had intracranial hemorrhages, and 36 (41.4%) were afflicted by other vascular entities. In the group suffering from inflammatory diseases, in total 41 (14.0%) patients can be allocated, with subgroups of 26 (63.4%) patients suffering from viral or bacterial meningitis, 11 (26.8%) patients inflicted by Guillain-Barré Syndrome, 1 patient with multiple sclerosis, and 3 patients with a neuromuscular disease (all myasthenia gravis). 73 nonventilated patients (25.0%) were admitted due to epilepsy, 6 patients (2.1%) were afflicted by degenerative diseases, and 85 patients (29.1%) were committed for other reasons, mostly intoxications or exacerbation of other neurological diseases due to nonneurological diseases. In the subgroup of ventilated patients, 96 (47.8%) patients had a cerebrovascular reason for insufficient respiration with an equal location in anterior/supratentorial (34.4%) and vertebrobasilar (33.3%) arterial circulation. Inflammatory diseases were diagnosed in 46 cases (22.8%), followed by epileptic genesis (12.9%).

### 3.2. Age

Mean age of nonventilated patients was 66 years; ventilated patients were 8 years older, on average. For nonventilated patients, age varies over the patient collectives, with patients suffering from inflammatory diseases having a median of 54 years, epilepsy with a median of 51 years, patients with vascular diseases with a median of 69 years, and degenerative diseases with a median of 67.5 years.

### 3.3. Length of Stay

**Figure 2:**
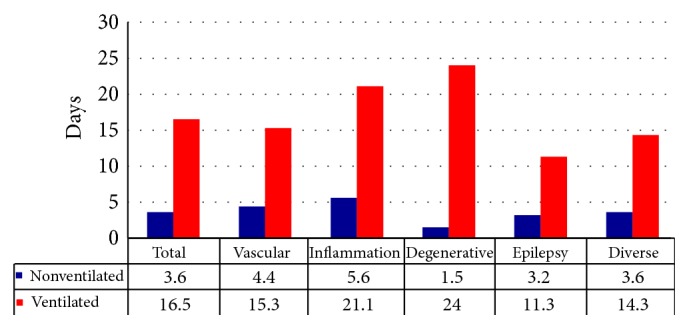
Length of stay (LOS).

For the group of nonventilated patients, mean LOS in our nICU was 3.6 days (range 1 to 6.4 days). These numbers differ between the different groups of patients in a low range: patients of the disease group of epilepsy (mean LOS 2.0 days) had a shorter length of stay than patients suffering from vascular (mean LOS 4.4 days) or inflammatory disease (mean LOS 5.6 days). Ventilated patients stayed 16.5 days in median, also strongly depending on indication for nICU treatment as shown in Figure 2.

### 3.4. Diagnostic Procedures

**Figure 3:**
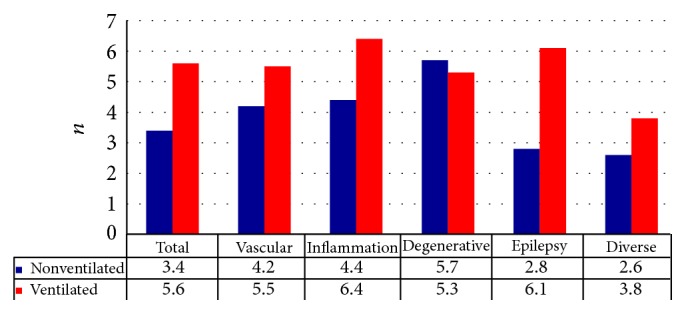
Diagnostic procedures/patient.

In total, the 493 patients included in the analysis underwent 2107 diagnostic procedures (4.2/patient). Thereby, on average, nonventilated patients were submitted to 3.4 procedures, while ventilated patients underwent 5.4 procedures. The subgroup of nonventilated patients with neurodegenerative (mean 5.6), inflammatory (mean 4.5), and vascular diseases (mean 4.2) went through more procedures than average, contrary to patients with epilepsy (mean 2.8). Ventilated patients had an average of 5.4 diagnostics/patient, with the highest rates of diagnostic investigation in the group of patients with inflammatory (6.3) or epileptic (6.1) diseases as shown in Figure 3.

### 3.5. Complications

**Figure 4:**
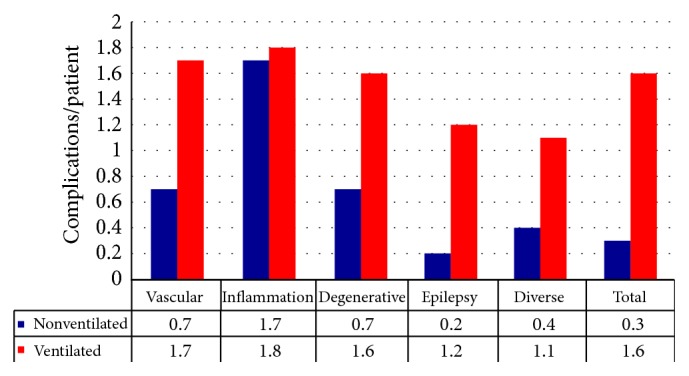
Complications/patients.

In the subgroup of nonventilated patients, in 33.2% of cases any kind of complications was seen, mostly neurological symptoms and aftereffects (16.4%), while secondary infections as a complication occurred in 12.0% of those cases. In the main group of patients with neurovascular diseases, 65.9% suffered from complications in general, especially with cardiac, respiratory, and neurological causes. Complications in the subgroup of ventilated patients were more common (87%), whereby infections (61.1%) and respiratory complications (54.7%) occurred most frequently as shown in Figure 4.

## 4. Discussion

The current study characterizes the diagnosis, treatment, and length of stay of patients in a stand-alone neurological intensive care unit between 2006 and 2008. As critical neurological patients collective have an overlap with neighbored disciplines like neurosurgery, internal medicine, and anesthesiology, treatment concepts and guidelines for same diseases differ between these specialties. In Germany, 36 independent nICUs are provided, mostly integrated in large hospitals and in direct contact to other clinical departments. Next to all advantages of these close bonds, from an economical and medical view evaluation of pure neurological data remains difficult. In the current study, a total of 493 patients were included, and more than every third patient (37.1%) had to be treated because of an acute neurovascular disease. This result is in line with studies from Broessner et al. and Harms et al. and reflects nICU crossover to stroke unit patients with acute ischemic stroke [5, 6]. Kiphuth et al. found cerebrovascular reasons in 60% of all patients treated in nICU, however, in a university clinic with a neurosurgical ICU next door [7]. Indications for transfer from stroke unit to nICU are given mostly in impaired consciousness, respiratory or cardiopulmonary complications, and endovascular embolectomy. As data show, stroke is the leading cause for nICU treatment and indirectly reflects the increasing need for nICU treatment opportunities especially in this subgroup. Until today only four Class I evidence based treatment options exist, treatment on stroke unit, intravenous tissue plasminogen activator within 4.5 h of stroke onset, decompressive craniotomy in malignant middle cerebral artery infarction, and aspirin within 48 h of stroke onset [8, 9]. Currently, four positive studies for endovascular embolectomy in large cerebral artery occlusion have lifted this treatment option in this highest category, but the need for general anesthesia and subsequently nICU options differs in the studies (9% in the ESCAPE trial, 38% in the MR Clean trial, 36% in EXTEND-IA, and 37% in SWIFT-PRIME).

Further management principles focus on hemodynamic and respiratory optimization next to control and treatment of fever, infection, glucose level [10], anticoagulation, antiplatelet, postinterventional management, and thromboprophylaxis. In the future, with regard to the increasing treatment options of acute neurological patients extended nICU settings will be needed.

Varelas et al. found a positive influence on lethality outcome and length of stay of neurological/neurosurgery patient if a specialized neurological setting is given [11]. In addition, an increasing LOS and length of mechanical ventilation are associated with poorer prognosis in longtime follow-up [5, 7]. The aim of neurointensive critical clinical care should also be to reduce duration of ventilation and length of stay. In 131 patients Vacca et al. described factors influencing the length of hospitalization in intensive care unit, which shows that sepsis as complication has the greatest impact, also treatment of infection is an important variable to reduce LOS [12]. In our cohort 33.2% of nonventilated and 87.0% of ventilated patients suffer from a complication, mostly respiratory infection, respectively, associated with mechanical ventilation. Ventilated patients have on average only 1.9 more diagnostic procedures as compared to nonventilated but a more than five longer LOS and clearly more complications like infections of the respiratory system.

In particular, in neurological patients with expected prolonged mechanical ventilation, another important variable to reduce LOS is time point of tracheostomy. Combes et al. postulate lower in-hospital and ICU mortality rates by early tracheostomy [13]. Shan et al. describe reduced mechanical ventilation duration if early tracheotomy is performed between third and seventh days in selected patients with expected prolonged ventilation duration [14]. The SETPOINT-study found early tracheostomy (days 1–3 versus days 7–14) in ventilated nICU stroke/hemorrhage patients is a safe and feasible method as part of weaning process and presumably reduces sedation [15]. Due to the diversity of nICU patients including their diversity of specific diseases a conclusion of a general time point of tracheostomy as part of weaning process cannot be drawn.

As the data reflects, relatively younger patients suffer from diseases like encephalitis or meningitis. Mostly patients are older than about 65 years and often have more than one complicating risk factor. The percentage of ventilated patients is lower, compared to a general or anesthesiological ICU (total 40.8%). Steffling et al. described in the same cohort that the most relevant indication for intubation was respiratory insufficiency in 32%. Mortality during stay on nICU was 15.4% (31/512) overall, and further 18.8% (32/170) of all survivors died during two months after discharging [16]. In terms of increasing diagnostic and therapeutic opportunities these results also suggest a need for standardized trials in nICU treatment for further reduction of complication rate, during ventilation and length of stay [17]. Effects of these new opportunities will be evaluated in an ongoing follow-up study.

## 5. Limitations

The current study has several limitations, including its retrospective nature and the fact that it is based on a single-center neurological intensive care unit with lack of neuro- and vascular surgeons. Furthermore, in the focused period interventional procedures just came up in our department and were not part of daily routine. Due to an overlap in personnel and technical resource for intensive care medicine and associated general neurology, this might have resulted in an increase of LOS and might have had an influence on the economical side.

## 6. Conclusion

Our study confirms the close relationship of cerebrovascular diseases and specific neurological intensive care treatment. Long-term ventilated patients require less diagnostic procedures/day and further studies should investigate the economical versus medical balance. Furthermore, a follow-up study might reveal the impact of neurointerventional treatment and their complications.

## 7. Outlook

The spectrum of possibilities for neurological treatment is changing rapidly. More options in therapy and diagnostic are available today, especially in diseases with raising incidence like autoimmune-moderated diseases (anti-NMDA-receptor encephalitis, LES) or inflammatory diseases (NMO, ADEM) [18]. Probably coming up new indications for immunoadsorption and plasmapheresis treatment are extending and are increasingly implemented as standard procedures in nICU, such as hypothermia in therapy of refractory status epilepticus or stroke [19, 20]. This retrospective and descriptive study gives basic data of treated patients in a stand- alone neurological intensive care unit. In a follow-up study data up to 2012 should compare subgroups and focus on time point of tracheostomy and might reflect progress in complex interventional neurological therapies.

## Figures and Tables

**Table 1:** Comparison of ventilated versus nonventilated patients and different diseases.

Disease	Ventilated			Nonventilated		
	[*n*]	Age (years) mean	Days ICU median	[*n*]	Age (years) mean	Days ICU median
Vascular	96	72	15	87	69	4.4
ICA/MCA/ACA	33	71	16	21	73	4.9
va/ba	32	74	18	10	55	3.8
ICH	24	63	12	20	67	4.4
Diverse	7	76	10	36	71	3.4
Inflammation	46	58	21	41	54	5.6
Viral/bacterial	18	32	20	26	51.5	5.7
GBS	7	68	18	11	66	6.4
Diverse (incl. MS, LES)	12	66	19			11
Neuromuscular	9	76	27	3	45	3
Degenerative	7	69	24	6	67.5	1.5
Hereditary	3	39	35	0	0	0
Epilepsy	26	55	11	73	51	2.0
Diverse	23	57	14	85	48	3.2
Total	201	66	16.5	292	58	3.6

**Table 2:** Procedures/pat./day.

	Patient total [*n*]	Length of stay (mean)	Procedures	Procedures/patient	Proc./pat./d
Nonventilated	292	3.6	1008	3.4	0.94
Ventilated	201	16.5	1099	5.4	0.34

**Table 3:** Diagnostic procedures in ventilated patient group.

Disease	Ventilated										
	Patient [*n*]	Diagnostic									
		Total	CCT	CT-Thorax	CT-Angio	cMRI	EEG	Ephys	Echo	Neurosono	Diverse
Vascular	96	521	164	103	46	24	32	16	47	63	26
ICA/MCA/ACA	33	184	57	43	9	4	14	3	22	19	13
va/ba	32	210	56	38	24	16	11	12	14	28	11
ICH	24	98	38	18	9	4	6	1	8	12	2
Diverse	7	29	13	4	4	—	1	—	3	4	—
Inflammation	46	294	47	70	10	22	40	40	27	24	17
Viral/bacterial	18	130	27	23	5	14	25	5	10	14	7
MS	2	11	1	5	—	—	1	1	1	1	—
GBS	7	40	6	5	2	2	7	14	1	1	2
Neuromuscular	9	54	3	22	1	1	—	11	7	4	5
Degenerative	7	37	6	10	—	7	9	3	2	—	—
Hereditary	3	5	1	3	—	—	—	—	—	1	—
Epilepsy	26	159	30	25	4	15	59	4	6	15	1
Diverse	33	142	30	37	4	10	20	10	14	14	—
Total	201	1099	268	233	64	73	152	64	88	113	44

**Table 4:** Diagnostic procedures in nonventilated patient group.

Disease	Nonventilated											
	Patient [*n*]	Diagnostic										
		Total	CCT	CT-Thorax	CT-Angio	cMRI	EEG	Ephys	Echo	DSA	Neurosono	Diverse
Vascular	87	369	95	26	21	46	18	5	68	9	51	30
ICA/MCA/ACA	21	99	31	10	3	11	3	1	21	1	15	3
va/ba	10	48	10	2	3	6	5	2	9	0	6	5
ICH	20	79	29	7	4	8	3	0	8	5	9	6
Diverse	36	143	25	7	11	21	7	2	30	3	21	16
Inflammation	41	184	19	19	3	29	20	22	10	0	8	54
Viral/bacterial	30	139	17	15	3	27	17	3	9	0	7	41
MS	1	4	1	0	0	0	1	0	0	0	0	0
GBS	11	40	1	4	0	1	2	18	1	0	1	12
Myasthenia gravis	3	1	0	0	0	0	0	1	0	0	0	0
Degenerative	6	34	4	2	2	4	4	5	2	0	1	10
Epilepsy	73	204	52	10	4	25	70	0	12	0	11	20
Diverse	85	217	50	33	11	18	31	7	17	1	18	33
Total	292	1008	220	90	41	121	143	39	109	10	89	146

## Appendix

See Tables 3 and 4.

## Abbreviations

nICU: Neurological intensive care unit
LOS: Length of stay
ICA: Internal carotid artery
va/ba: Vertebral artery/basilar artery
ICH: Intracerebral hemorrhage
GBS: Guillain-Barré Syndrome
MS: Multiple sclerosis
LES: Lambert-Eaton Syndrome
NMDA: N-Methyl-D-aspartate
NMO: Neuromyelitis optica
ADEM: Acute disseminated encephalomyelitis.

## References

[1] Deuschl G., Müllges W. (2007). Stellungnahme der Deutschen Gesellschaft für Neurologische Intensiv-und Notfallmedizin (DGNI) und der Deutschen Gesellschaft für Neurologie (DGN) zur Neurologischen Intensivmedizin. *Aktuelle Neurologie*.

[2] Suarez J. I., Zaidat O. O., Suri M. F. (2004). Length of stay and mortality in neurocritically ill patients: impact of a specialized neurocritical care team. *Critical Care Medicine*.

[3] Varelas P. N., Eastwood D., Yun H. J. (2006). Impact of a neurointensivist on outcomes in patients with head trauma treated in a neurosciences intensive care unit. *Journal of Neurosurgery*.

[4] Shan L., Zhang R., Li L.-D. (2013). Effect of timing of tracheotomy on clinical outcomes: an update meta-analysis including 11 trials. *Chinese Medical Sciences Journal*.

[5] Broessner G., Helbok R., Lackner P. (2007). Survival and long-term functional outcome in 1,155 consecutive neurocritical care patients. *Critical Care Medicine*.

[6] Harms L., Garner C., Einhaupl K. M. (1998). The status of neurologic intensive care in Germany. Situation der neurologischen Intensivmedizin in Deutschland. *Der Nervenarzt*.

[7] Kiphuth I. C., Schellinger P. D., Köhrmann M. (2010). Predictors for good functional outcome after neurocritical care. *Critical Care*.

[8] Donnan G. A., Fisher M., Macleod M., Davis S. M. (2008). Stroke. *The Lancet*.

[9] Lees K. R., Bluhmki E., von Kummer R. (2010). Time to treatment with intravenous alteplase and outcome in stroke: an updated pooled analysis of ECASS, ATLANTIS, NINDS, and EPITHET trials. *The Lancet*.

[10] Kruyt N. D., Biessels G. J., Devries J. H., Roos Y. B. (2010). Hyperglycemia in acute ischemic stroke: pathophysiology and clinical management. *Nature Reviews Neurology*.

[11] Varelas P. N., Conti M. M., Spanaki M. V. (2004). The impact of a neurointensivist-led team on a semiclosed neurosciences intensive care unit. *Critical Care Medicine*.

[12] Vacca F., Vaiani M., Messori A. (2004). Factors influencing the length of hospitalisation in intensive care units: a prospective observational study. *Pharmacy World and Science*.

[13] Combes A., Luyt C.-E., Nieszkowska A., Trouillet J. L., Gibert C., Chastre J. (2007). Is tracheostomy associated with better outcomes for patients requiring long-term mechanical ventilation?. *Critical Care Medicine*.

[14] Shan L., Hao P., Xu F., Chen Y.-G. (2013). Benefits of early tracheotomy: a meta-analysis based on 6 observational studies. *Respiratory Care*.

[15] Bösel J., Schiller P., Hook Y. (2013). Stroke-related early tracheostomy versus prolonged orotracheal intubation in neurocritical care trial (SETPOINT): a randomized pilot trial. *Stroke*.

[16] Steffling D., Ritzka M., Jakob W. (2012). Indications and outcome of ventilated patients treated in a neurological intensive care unit. *Nervenarzt*.

[17] Kowoll C. M., Dohmen C., Kahmann J. (2014). Standards of scoring, monitoring, and parameter targeting in German neurocritical care units: a national survey. *Neurocritical Care*.

[18] Kim S.-H., Kim W., Huh S.-Y., Lee K. Y., Jung I. J., Kim H. J. (2013). Clinical efficacy of plasmapheresis in patients with neuromyelitis optica spectrum disorder and effects on circulating anti-aquaporin-4 antibody levels. *Journal of Clinical Neurology*.

[19] Kollmar R., Schwab S. (2012). Hypothermia and ischemic stroke. *Current Treatment Options in Neurology*.

[20] Wu T.-C., Grotta J. C. (2013). Hypothermia for acute ischaemic stroke. *The Lancet Neurology*.

